# BCG Revaccination for the Prevention of *Mycobacterium tuberculosis* Infection

**DOI:** 10.1056/NEJMoa2412381

**Published:** 2025-05-08

**Authors:** Alexander C Schmidt, Lee Fairlie, Elizabeth Hellström, Angelique Luabeya Kany Kany, Keren Middelkoop, Kogieleum Naidoo, Gonasagrie Nair, Anele Gela, Elisa Nemes, Thomas J Scriba, Amy Cinar, Nicole Frahm, Robin Mogg, David Kaufman, Michael W Dunne, Mark Hatherill, the BCG REVAX Study team

**Affiliations:** 1 Bill & Melinda Gates Medical Research Institute, Cambridge, MA, USA; 2 Wits RHI, University of Witwatersrand, Johannesburg, GP, South Africa; 3 Be Part Yoluntu Centre NPC, Paarl, WC, South Africa; 4 South African Tuberculosis Vaccine Initiative, Institute of Infectious Disease and Molecular Medicine and Department of Pathology, University of Cape Town, Cape Town, WC, South Africa; 5 The Desmond Tutu HIV Centre, University of Cape Town, Cape Town, WC, South Africa; Institute of Infectious Disease and Molecular Medicine, University of Cape Town, Cape Town, WC, South Africa; 6 CAPRISA MRC-HIV-TB Pathogenesis and Treatment Research Unit, Centre for the AIDS Programme of Research in South Africa, Durban, South Africa

## Abstract

**BACKGROUND:**

In a prior phase 2 trial (NCT02075203), BCG revaccination failed to provide protection from primary *Mycobacterium tuberculosis* (*Mtb*) infection but prevented sustained *Mtb* infection (secondary endpoint), defined as initial interferon gamma release assay (IGRA) conversion followed by two additional positive IGRA results 3 and 6 months post initial conversion, with a vaccine efficacy [VE] of 45% (95% CI 6% to 68%).

**METHODS:**

This randomized, double-blind, placebo-controlled, phase 2b trial evaluated the safety, immunogenicity, and efficacy of BCG revaccination for the prevention of sustained IGRA conversion in IGRA-negative, HIV-negative adolescents. VE was evaluated in the modified intention-to-treat (mITT) set that required a negative IGRA test 10 weeks post receipt of trial treatment to exclude participants with *Mtb* infection around the time of vaccination. Hazard ratio (HR) and 95% CIs were estimated from a stratified Cox proportional hazards model.

**RESULTS:**

A total of 1836 participants were enrolled; 918 participants received BCG and 917 received placebo. 121 cases of sustained IGRA conversion were observed in the mITT set after a median 30 months of follow-up: 62 cases among 871 BCG recipients and 59 among 849 placebo recipients. The observed HR was 1.038 (95% CI 0.73 to1.48), for a VE point estimate of −3.8% (95% CI −48.3% to 27.4%). Adverse events were more frequent in the BCG group, mostly due to injection site reactions (pain, redness, swelling, ulceration). BCG revaccination induced Th1-cytokine-positive CD4 T cells.

**CONCLUSIONS:**

BCG revaccination of IGRA-negative adolescents does not provide protection from sustained *Mtb* infection. (Supported by Bill & Melinda Gates Foundation; ClinicalTrials.gov Identifier NCT04152161.)

Approximately 1.7 billion individuals are estimated to be infected with *Mycobacterium tuberculosis* (*Mtb*) globally, of whom about 56 million are newly infected and at high risk of progression to tuberculosis disease (TB)^1^. In 2022, an estimated 10.6 million new cases of TB were reported worldwide, including 1.3 million children <15 years old^2^. Globally, TB was responsible for 1.3 million deaths in 2022, including 167,000 people living with human immunodeficiency virus (HIV)^2^. South Africa has one of the highest TB incidence rates in the world, estimated at 468/100,000 in 2022^2^. Cohort studies in the Western Cape province showed that the prevalence of *Mtb* infection, as assessed by an *Mtb*-specific interferon-gamma release assay (IGRA) such as QuantiFERON-TB tests (QFT), increases quickly in adolescence^3,4^, with QFT conversion rates as high as 10% per year^3^.

A prior phase 2 trial assessed the efficacy of the investigational H4:IC31 vaccine and Bacille Calmette-Guérin (BCG) vaccine versus placebo for protection against *Mtb* infection in healthy 12–17-year-old IGRA-negative, HIV-negative participants in South Africa^3^. BCG revaccination failed to provide protection from primary *Mtb* infection, assessed by initial QFT conversion. However, significant vaccine efficacy (VE) was observed for the secondary endpoint, prevention of sustained *Mtb* infection (POSI; VE 45%, 95% CI 6% to 68%), defined as initial QFT conversion followed by positive QFT results at 3 and 6 months thereafter^3^.

One interpretation of the significance of prevention of sustained *Mtb* infection is that QFT reversion (from QFT-positive to QFT-negative) could indicate successful immune control or clearance of *Mtb* infection, thus decreasing the risk of progression to disease^5^. However, because no more than 5-10% of *Mtb*-infected individuals progress to TB, VE for prevention of sustained *Mtb* infection may not predict VE for prevention of disease (POD) if the protective immune response leading to prevention of sustained *Mtb* infection is only induced in the majority of individuals who would not have progressed to disease without vaccination^6^.

In the absence of a licensed vaccine for the prevention of TB in adolescents and adults, we set out to: a) confirm that BCG revaccination confers prevention of sustained *Mtb* infection in a larger population with expanded age range and geographical area; and b) identify candidate correlates of protection for this trial endpoint, assuming that a confirmatory trial could potentially support a BCG revaccination policy change in South Africa, or motivate funders to invest in a large BCG phase 3 POD trial, in support of the global effort to accelerate the end of the TB epidemic^7,8^. Here we report the results of a phase 2b trial to assess the VE of BCG revaccination for prevention of sustained *Mtb* infection in South Africa.

## Methods

### Trial Design and Objectives

This randomized, placebo-controlled, observer-blind, phase 2b trial enrolled participants at 5 sites in South Africa: Worcester, Cape Town, and Mbekweni in Western Cape; Durban in KwaZulu-Natal; and Johannesburg in Gauteng Province. The primary objective was to demonstrate the efficacy of BCG revaccination against sustained *Mtb* infection versus placebo in QuantiFERON^®^-TB-Gold-Plus assay (QFT)-negative, healthy adolescents.

Secondary objectives were to evaluate the durability of efficacy, and safety and reactogenicity of BCG revaccination. Exploratory objectives included the immunogenicity of BCG revaccination, VE based on alternative definitions of QFT conversion, and incident cases of laboratory-confirmed TB.

**Figure 1:**
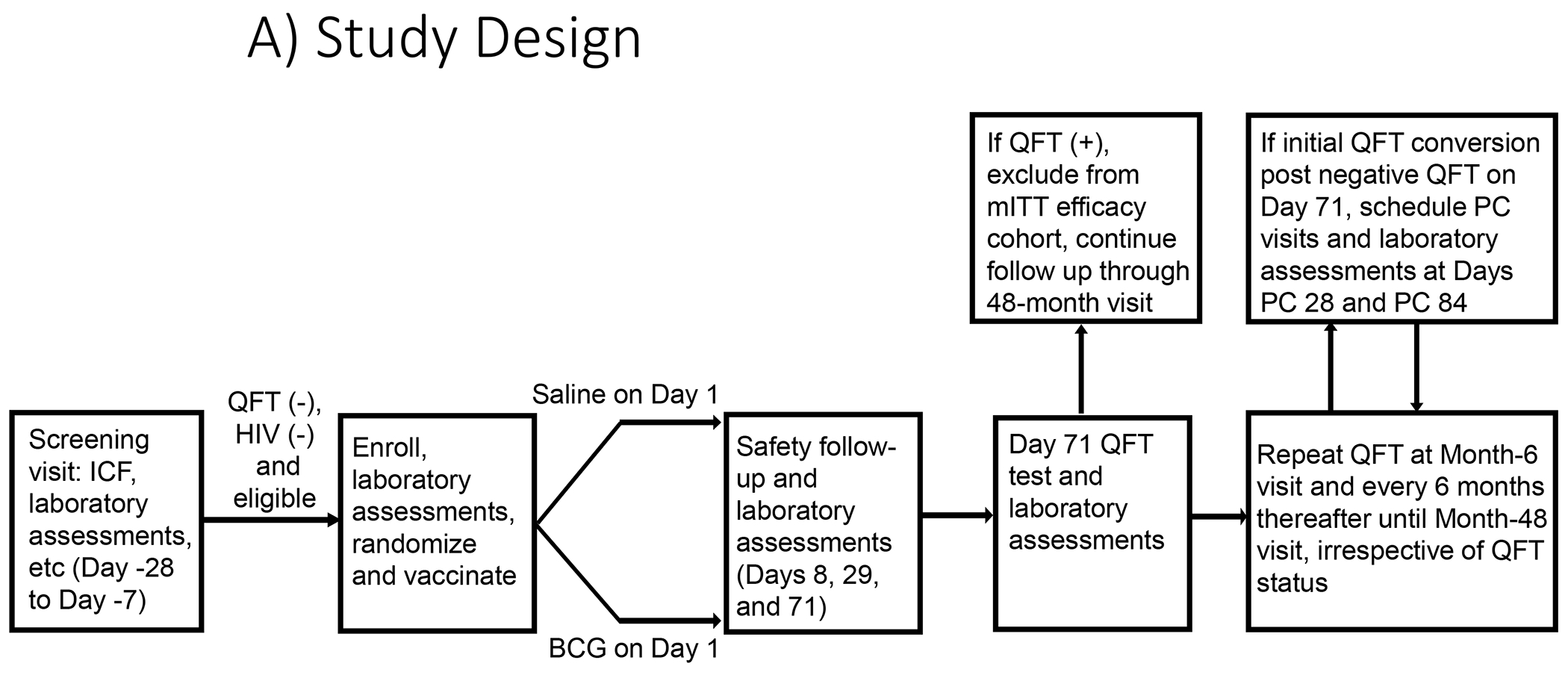

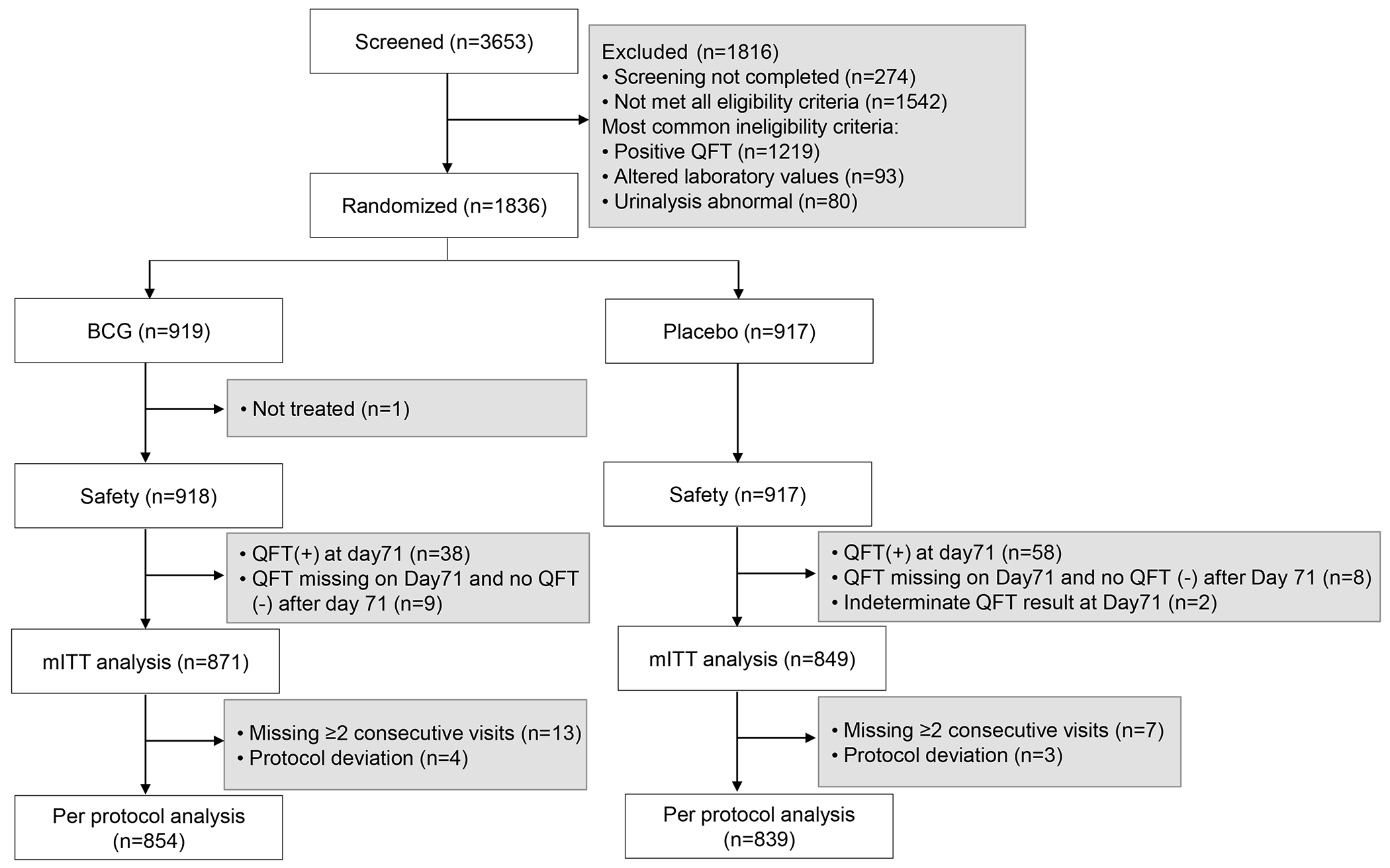
Trial Design and CONSORT diagram A) Trial Design. Participants were randomized to placebo or BCG groups and QFT assessments were performed according to trial schedule. Participants who converted to QFT positive at Day 71 (or if missed or not feasible, at the next feasible visit) were excluded from the primary mITT efficacy analysis but were followed for safety and efficacy to end of study. Each participant was followed for safety for a minimum of 6 months after vaccination. ICF, informed consent form; PC, post conversion; QFT, QuantiFERON-TB Test B) Participant Disposition. Amongst 3653 screened individuals, 1816 were excluded from enrollment. The most common ineligibility criteria were a positive QFT test, altered laboratory values, urinalysis abnormal. Protocol deviations leading to exclusion from mITT population included IP non-compliance (3 in the BCG group) and eligibility criteria (1 in the BCG group and 3 in placebo). A total of1836 were randomized 1:1 to BCG (n=919) or placebo (n=917). IP, Investigational product; QFT, QuantiFERON-TB Test

Participants were randomized 1:1 to receive BCG (BCG Danish 1331 vaccine, AJ Vaccines, Copenhagen, Denmark) or placebo intradermally (Figure 1). Since BCG does not include antigens used in the QFT assay, revaccination is not associated with QFT conversion and negative QFT result at Day (D) 71 could be used to define the mITT population for VE assessment. Randomization was stratified by age, sex, trial site, and school cluster (Worcester site only). BCG was administered as per product package insert for South Africa (0.1mL intradermally). Randomization to BCG or placebo was assigned using a validated Interactive Voice/Web Response System. The trial was observer-blinded until the primary endpoint analyses were completed. Inadvertent unblinding occurred due to recognizable lesion formed at the BCG injection site.

### Participants

Eligible participants were ≥10 and ≤18 years old and tested negative for HIV and QFT. Detailed eligibility criteria are described in the Supplement and protocol at nejm.org.

### Outcomes and assessments

The primary endpoint was sustained QFT conversion based on positive QFT results using the manufacturer’s (Qiagen^®^, Germany) assay threshold of 0.35 IU/mL IFNγ. Sustained *Mtb* infection was defined as sustained QFT conversion from a negative to a positive test, with initial conversion at any time after a first negative QFT post randomization, and remaining QFT-positive at 3- and 6-months post conversion. Figure 1A shows the sampling schedule.

Secondary endpoints of safety and reactogenicity of BCG revaccination were evaluated based on the adverse events (AEs) reported as described in the supplement. Exploratory efficacy endpoints included initial QFT conversion using various IFN-γ thresholds ranging from 0.36 IU/mL to 10 IU/mL.

The first 80 10-12-year-old participants randomized at the Worcester site were enrolled in the immunogenicity sub-cohort since this younger age group had not previously been evaluated for immunogenicity. Immunogenicity was evaluated as the frequency of BCG-specific CD4 or CD8 T cells expressing one or more of the following cytokines: IFN-γ, tumor necrosis factor (TNF), interleukin (IL)-2, IL-17 and/or IL-22, by whole blood intracellular cytokine staining (WB-ICS) assay.

### Trial oversight

An independent data monitoring committee reviewed unblinded safety data every three months during enrollment and every six months thereafter, as well as the outcomes of the primary analyses. The trial was conducted in accordance with the International Council for Harmonization of Technical Requirements for Pharmaceuticals for Human Use (ICH) Good Clinical Practice (GCP) Guidelines, SAHPRA (South African Health Products Regulatory Authority) Regulations, the IRB/IEC, and other applicable country and local requirements. All authors contributed to data collection, data analysis, reviewed the manuscript, and vouch for the accuracy and completeness of the data presented.

### Statistical analysis

The trial was designed to provide 90% power with a 1-sided alpha of 2.5% for the primary endpoint analysis. Assuming a true VE of 45%, at least 118 sustained QFT conversion events were required to demonstrate VE with a lower bound of zero, where VE is calculated as 1 – HR(BCG/Placebo).

The modified intention-to-treat (mITT) population included all participants who received the trial treatment and were QFT negative at the D71 visit, or at the first trial visit post D71 for which a QFT result was available. The safety population included all participants who received the trial treatment. Frequency (n) and percentages (%) were used to summarize categorical variables; mean, standard deviation (SD), median, minimum, and maximum were used to summarize continuous variables.

The primary efficacy endpoint was analyzed using a log-rank test, stratified by sex and age group (10-11 years old, 12-14 years old, and >14 years old) to evaluate differences in distributions of event times between the BCG revaccination and placebo groups. AEs in different categories were summarized by treatment groups and the 95% CI were calculated for a single proportion using the mid-p binomial option. AEs were coded using Medical Dictionary for Regulatory Activities (MedDRA) version 26.0 or higher. AEs were graded using the Division of AIDS Table for Grading for Severity of Adult and Pediatric Adverse Events Version 2.1, July 2017^9^.

The percentage of missing data was low, with a discontinuation rate of 5.1% and 3.9% for the BCG and placebo groups, respectively (including 1.0% and 0.2% lost to follow up in the BCG and placebo groups and 2.5% participant withdrawal in each group). Given that the missingness was minimal and did not appear to be dependent on known factors, we assume the data to be missing completely at random and therefore, only the observed data were used in analyses.

## Results

### Baseline characteristics and disposition

From 16 Oct 2019 to 22 July 2021, 3653 participants were screened and 1836 were randomized 1:1 to BCG and placebo groups (Figure 1B). Table S1 shows the representativeness of trial participants. At primary analysis (data cutoff: June 30, 2023), 1752 participants were in trial follow-up and 83 (4.5%) discontinued. Among the 3653 screened participants, 307 had no blood drawn, 12 had indeterminate QFT results, and 1 had invalid results, and of the remaining 3333 participants with screening QFT results available, 1219 (36.6%) were QFT positive (Table S2). QFT positivity rates at screening were higher at sites in the Western Cape (37.8%-39.6%) than in KwaZulu-Natal (29.1%) or Gauteng (23.6%) provinces. Demographics and baseline characteristics were comparable between the two groups (Table S3). The median age was 13 years (range, 10-18) and most participants (1464 [79.8%]) self-identified as Black African.

### Efficacy

**Figure 2:**
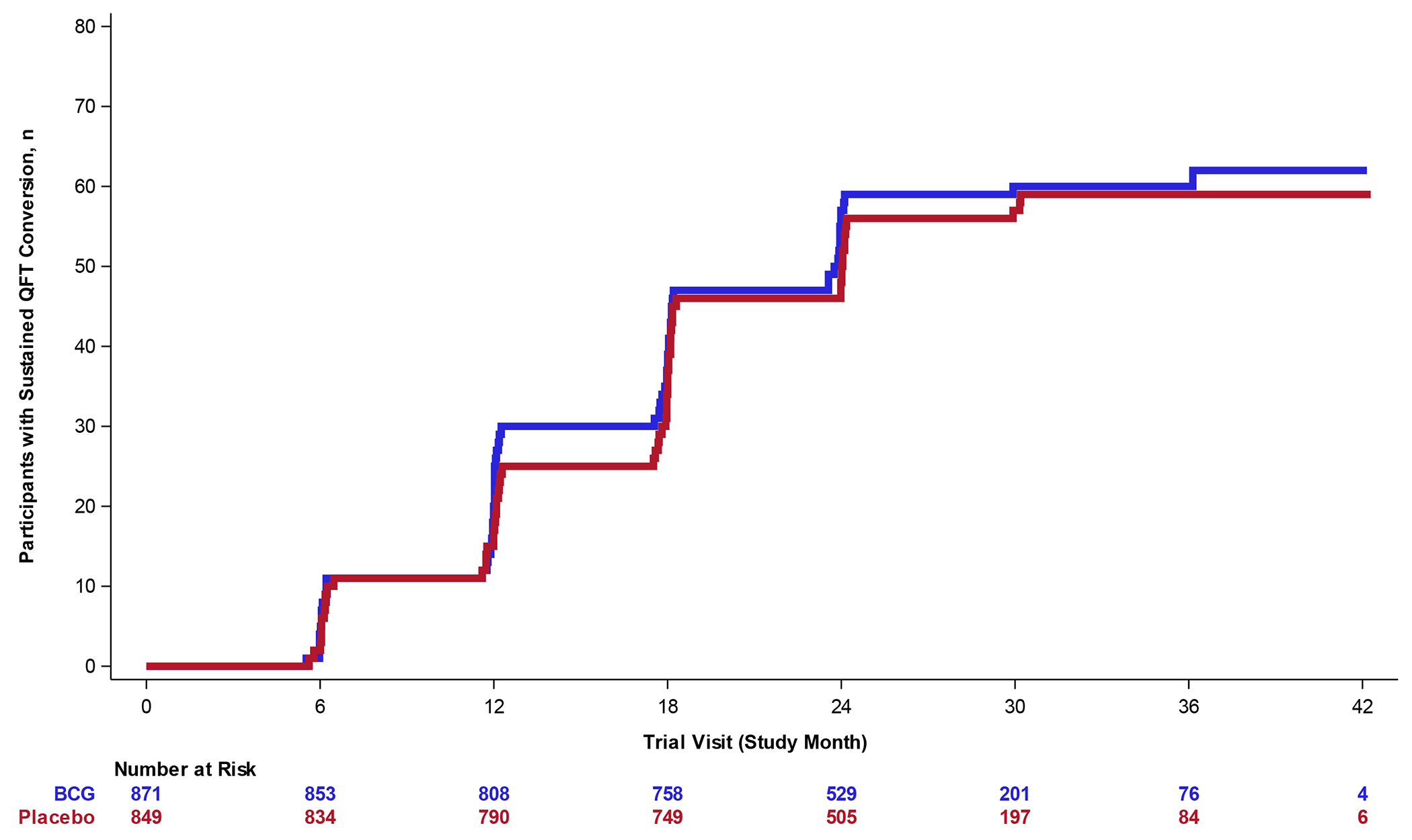

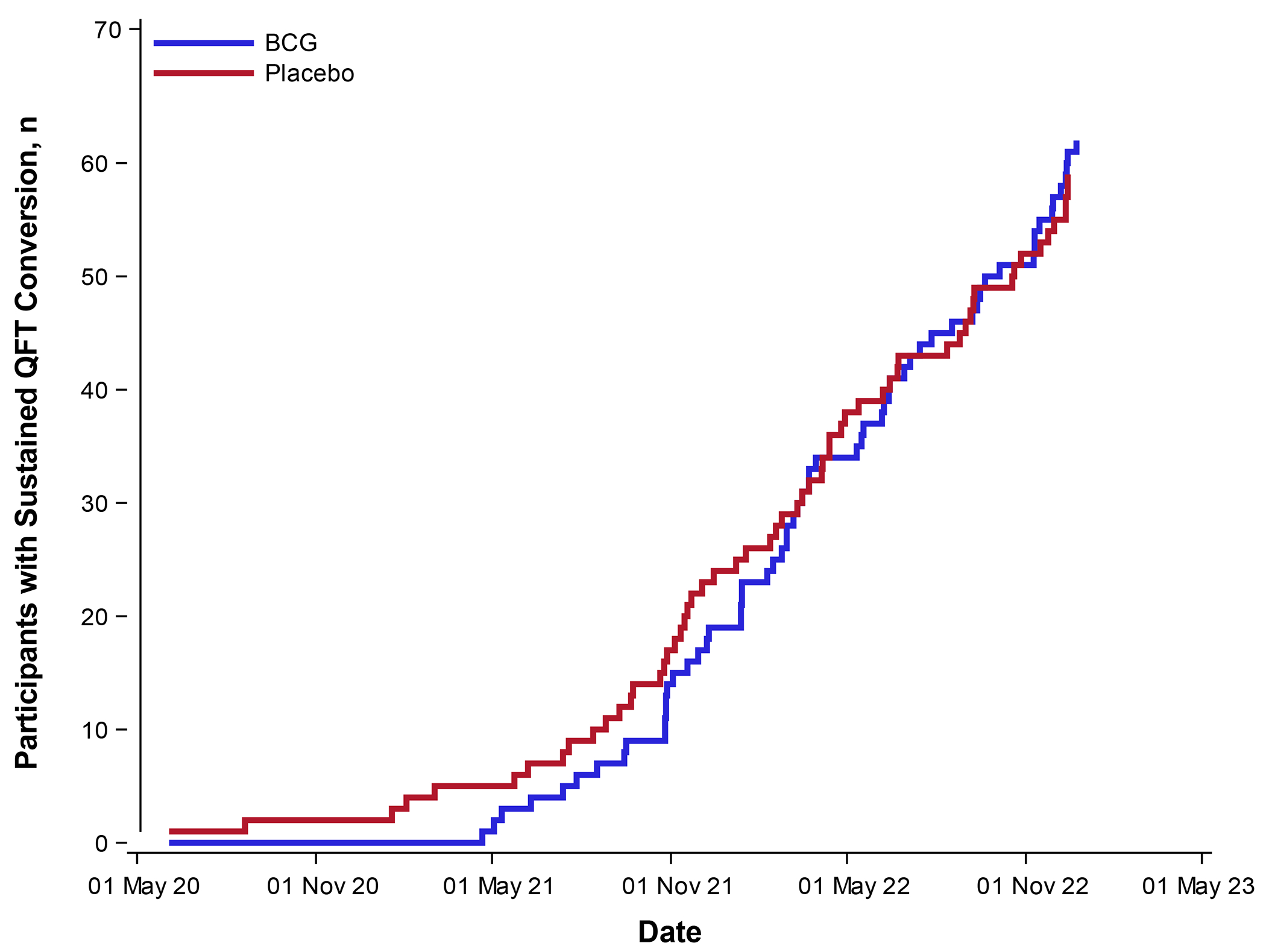

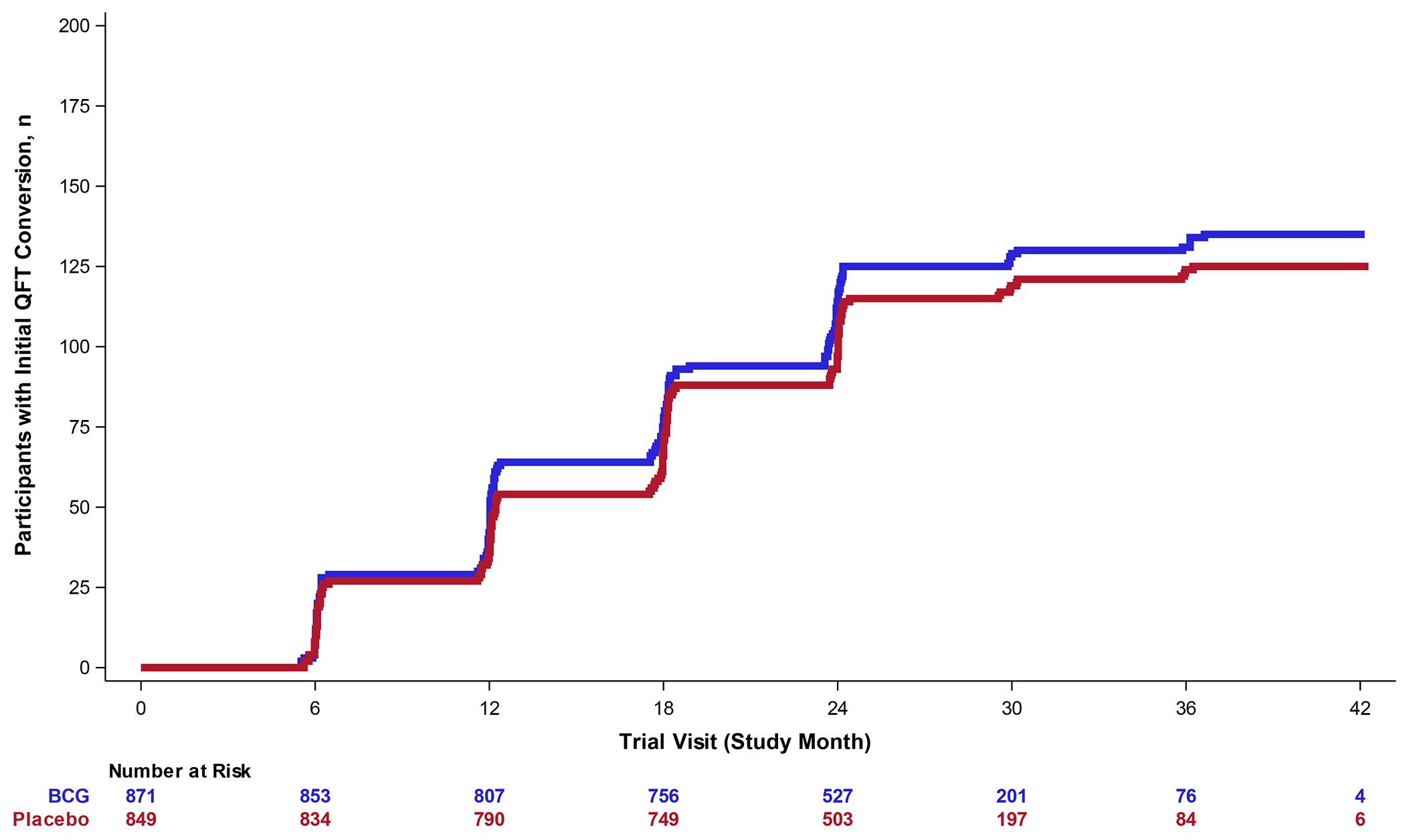

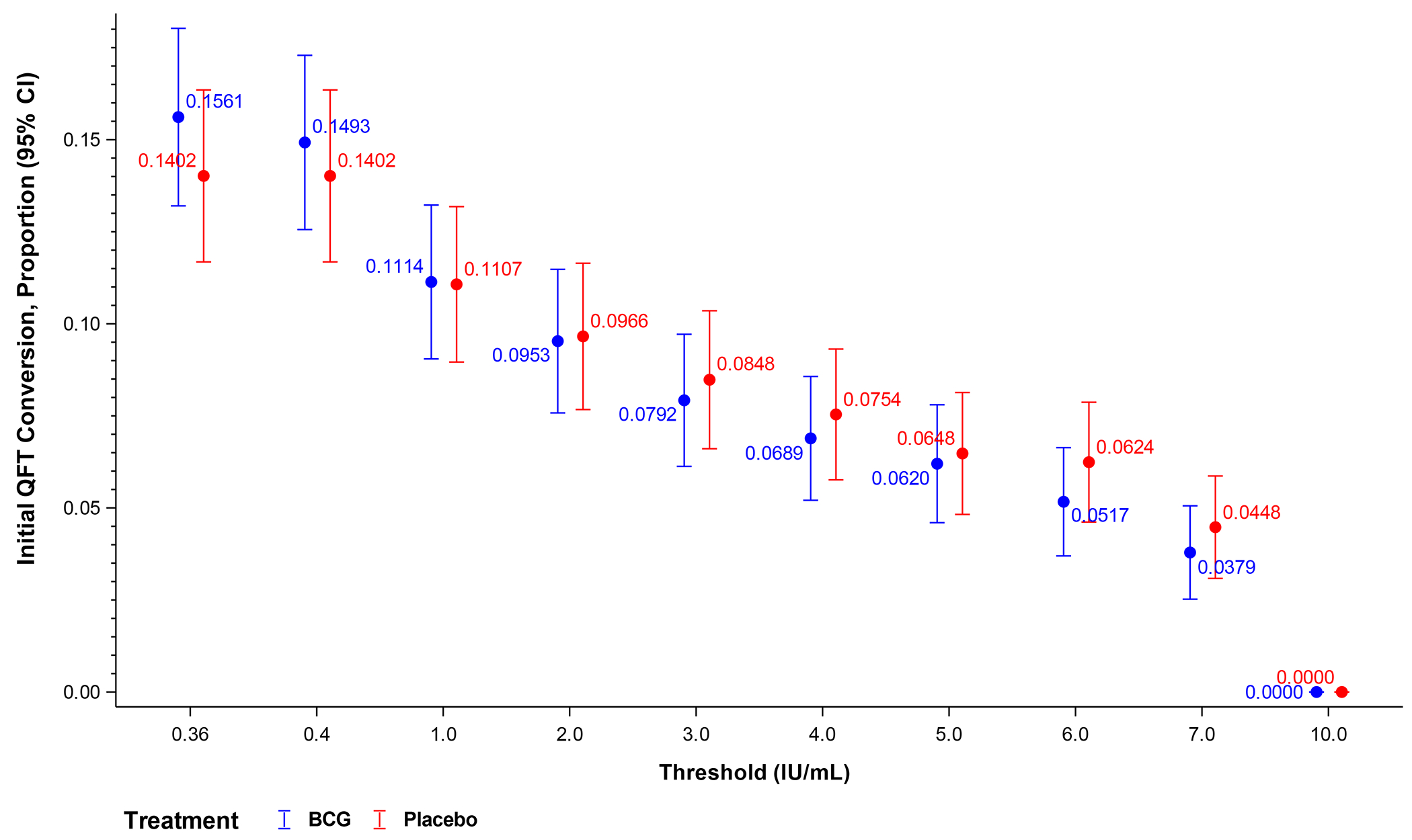
Vaccine Efficacy in the mITT population. A) Cumulative event curve for sustained QFT conversion (primary efficacy endpoint) by trial visit. B) Cumulative event curve for sustained QFT conversion by calendar month. C) Cumulative event curve for initial QFT conversion by trial visit. D) Proportion of initial QFT conversions based on QFT conversion thresholds of >0.35 IU/mL. Error bars represent 95% CIs. CI, confidence intervals; mITT, modified intention-to-treat population; QFT, QuantiFERON-TB Test

The mITT population included 871 and 849 participants in the BCG and placebo groups, respectively (Figure 1B). Thirty-eight (4.1%) and 58 (6.3%) participants were excluded from the mITT population due to positive QFT at D71; 9 (1.0%) and 8 (0.9%) were excluded due to missing D71 and no negative result at the first visit after D71; and 0 and 2 (0.2%) were excluded due to indeterminate results at D71. At primary analysis after a median of 30 months (interquartile range, 25 to 33) of follow up, sustained QFT conversion was observed in 62 participants in the BCG group and 59 in the placebo group (one-sided P=0.58; Table 1, Figure 2A & 2B). The sustained QFT conversion rate (95% CI) was 7.1% (5.4 to 8.8) in the BCG group and 7.0% (5.2 to 8.7) in the placebo group. The overall sustained conversion incidence rate and the incidence rate over time were similar in both groups (Table 1, Figure 2A & 2B). The VE point estimate for the primary endpoint was −0.038 (95% CI, −0.483 to 0.274) (Table 1). Site specific VE point estimates are listed in Table S4.

Overall, there were 135 (15.5%) initial QFT conversions (after D71) in the BCG group and 125 (14.7%) conversions in the placebo group (Table 1, Figure 2C). QFT reversion rates were similar in both groups (Table 1, Table S5). Amongst initial QFT converters with 3 consecutive positive or negative QFT results available, 62 of 111 BCG recipients (56%) and 59 of 101 placebo recipients (58%) developed sustained QFT conversion, while 49 (44%) and 42 (42%) developed QFT reversion at D84 or Month 6 post conversion.

The initial QFT conversion rates based on a range of increasingly stringent thresholds were similar between the BCG and placebo groups, regardless of threshold (Figure 2D, Table S6). For example, initial QFT conversions based on a QFT threshold of 4.0 IU/mL were observed in 60 (6.9%) participants in the BCG group and 64 (7.5%) in the placebo group (Figure 2D, Table S6, Figure S1).

Six participants in the safety population (3 in each of the BCG and placebo groups) developed laboratory-confirmed TB.

### Safety

Solicited local AEs were reported by 77.8% participants in the BCG group and 38.1% in the placebo group; 40.5% and 34.3% reported any solicited systemic AEs, respectively (Table 2). Swelling was the most common solicited local AE, and tiredness was the most common solicited systemic AE.

Unsolicited non-serious AEs were experienced by 185 (20.2%) participants in the BCG group and 117 (12.8%) in the placebo group (Table 2). The most common unsolicited non-serious AEs with BCG were headache (3.9%) and injection-site pain (2.3%). Most AEs were mild to moderate in severity. Three participants (0.3%) in each group experienced serious AEs, which were assessed by the site investigator as not related to trial intervention. No SAEs with the outcome of death, serious ADRs or AEs leading to premature trial discontinuation were reported. In the BCG group, 32 participants developed injection site scars >10mm. Of them, 9 were >15mm and 2 were >20mm. One larger keloid (2.5 cm max. diameter) was surgically removed at the participant’s request.

### Immunogenicity

**Figure 3:**
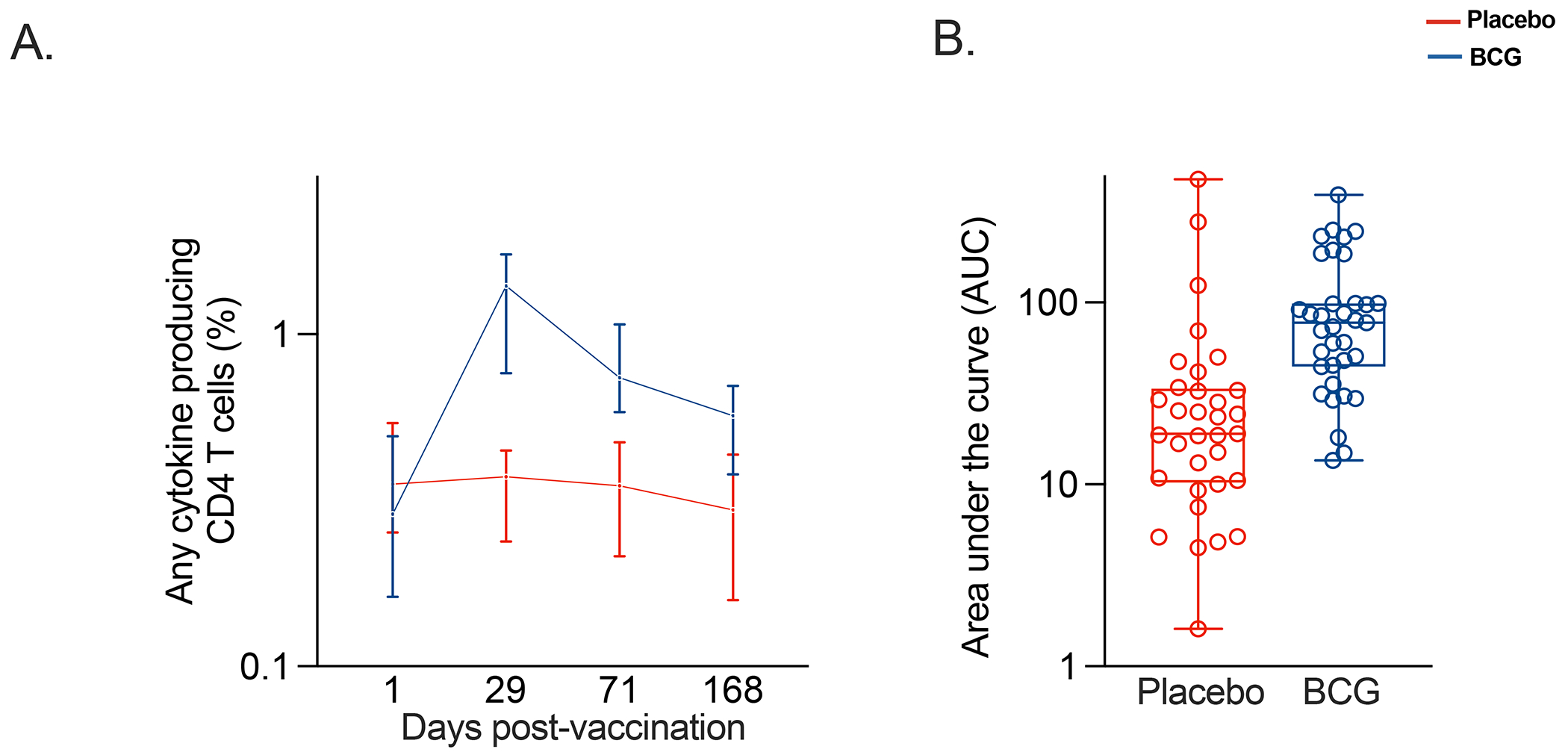
Immunogenicity of BCG revaccination. Frequencies of antigen-specific CD4 T cells expressing any combination of IFN-γ, TNF, IL-2, IL-17, and/or IL-22 after stimulation with BCG, measured by WB-ICS assay in participants receiving placebo (red) or BCG (blue). Panel A shows longitudinal responses during the first 168 days after vaccination. Lines represent medians and errors bars 95% CIs. Panel B shows area under the curve (AUC) for antigen-specific CD4 T cell response during the first 168 days after vaccination. Each dot represents an individual participant.

BCG revaccination increased the frequencies of antigen-specific CD4 T cells expressing any combination of IFN-γ, TNF, IL-2, IL-17, and IL-22, compared to placebo, which remained higher than baseline, 6 months after vaccination (Figures 3, S2 and S3). Th1 responses (CD4 T cells expressing IL-2, IFN-γ, and/or TNF) predominantly accounted for this observation, rather than IL-17 or IL-22 producing CD4 T cells (Figure S3). A transient increase of CD8 responses to BCG was observed at D29 post-vaccination (Figure S4).

## Discussion

This trial was conducted to assess the findings of an earlier trial that reported a significant reduction in sustained QFT conversion rates following BCG revaccination of QFT-negative, HIV-negative adolescents^3^, assuming that a confirmatory prevention of sustained *Mtb* infection result and supportive correlates of protection data could de-risk and motivate a future POD trial. While a prevention of sustained *Mtb* infection VE point estimate of 45.4% (95% CI, 6.4 to 68.1) was observed in the earlier trial, no efficacy was observed in the trial reported here (VE point estimate −3.8% [95% CI, −48.3 to 27.4]). The differences in observed efficacy are unlikely due to differences in BCG strain, case definition, biospecimen processing, or assay performance, which were intentionally harmonized. This trial differed from the previous trial in that it was powered for sustained QFT conversion as the primary endpoint using QFT TB Gold-Plus rather than the QFT TB Gold In-tube test; the sample size per treatment arm was approximately 3 times larger; the age range increased to include 10- and 11-year-olds; and the geographical footprint expanded to include additional communities in the Western Cape, Gauteng, and KwaZulu Natal provinces with a more diverse population.

However, geographical or ethnic differences between trials are unlikely to explain the observed differences in vaccine efficacy, since the observed site-specific VE in this trial at the Worcester site where 92.7% of the earlier trial was enrolled, was similar to the overall VE observed in this trial. The overall initial QFT conversion rate in this trial (6.5-6.9% per person-year) was lower than the 9.9% per person-year reported for the earlier trial. This could be due, in part, to the COVID-19-related lockdown with extended school-closures in 2020 and 2021^10^, and inclusion of trial sites with a lower force of infection: two sites outside the Western Cape province enrolled 20% of participants but only contributed 5.0% [6/121] of primary endpoint cases. One observation that differed between this trial and the earlier trial and may have contributed to the difference in observed VE is that the QFT reversion rate in the placebo arm was higher in our trial (42%) than the earlier trial (25%) while the reversion rates in the BCG group were comparable (44% vs 46%). Whether differences in baseline characteristics (inclusion of younger children, more diverse population) or lower intensity of *Mtb* exposure, e.g., related to COVID-19 lock-down restrictions, contributed to the observed difference in reversion rates remains unknown.

The safety and reactogenicity data reported here agrees with the known safety profile of the marketed BCG vaccine. This vaccine is well characterized. Larger scars (>10mm) at the site of BCG administration were observed in approximately 3% of BCG vaccinees, consistent with previous reports ^11^.

A BCG revaccination trial for prevention of sustained *Mtb* infection in 2,000 adult health-care workers in Brazil recently reported the absence of VE against either initial or sustained QFT conversion^12^. Given that two randomized controlled trials in settings with different force of infection, geography, climate, exposure to environmental mycobacteria, ancestry and age, observed a lack of VE, the evidence supporting BCG revaccination for the prevention of *Mtb* infection appears weakened. However, our data do not allow conclusions to be drawn on the potential efficacy of BCG revaccination for POD, for which supporting evidence is limited.^13,14^ A BCG revaccination trial for POD is underway in India and may provide informative data on that endpoint^15^.

Significant attention has been given to the predictive power of the QFT assay for TB disease, and higher IFN-γ conversion values were previously reported to be associated with a higher risk of progression to TB^13,14^. However, for POI, we observed no changes in VE with increasing IFN-γ thresholds, arguing against the possibility that false-positive converters contributed to the observed lack of VE.

The strengths of this dataset include that this trial employed stringent statistical criteria to assess VE, and that the primary endpoint data are unambiguous. BCG revaccination did not provide any protection from *Mtb* infection as assessed by QFT, whether defined as sustained conversion, initial conversion, or conversion with higher IFN-γ thresholds. Treatment groups were well balanced, and the trial was well executed, with stringent monitoring and oversight. Participant retention and community engagement were excellent throughout.

While this trial does not allow us to draw firm conclusions on the efficacy of BCG revaccination for POD, the absence of VE for POI probably decreases the likelihood of BCG revaccination conferring POD, unless by prevention of progression from infection to disease. Other limitations include that enrollment was paused for four months due to COVID-19 pandemic restrictions, and schools were closed for several additional months^10^, which may have contributed to a lower incidence rate.

## Supplementary Material

Supp

## Figures and Tables

**Table 1.** Sustained and initial QFT Conversions*

Variable	BCG (N=871)	Placebo (N=849)
Initial QFT converter (on or after Day 71), n (%)^††^	135 (15.5)	125 (14.7)
Not evaluable for QFT reversion (missing or indeterminate QFT results)	24	24
QFT reversion at Day 84 or Month 6 PC, n (%)	49	42
Participants with sustained conversion, n (proportion) [95% CI] ^‡^	62 (0.0712) [0.0541 to 0.0883]	59 (0.0695) [0.0524 to 0.0866]
Person time accrued (months)^¶^	19719.2	19383.2
Overall sustained conversion incidence rate (95% CI)^¶¶^	0.00314 (0.00241 to 0.00403)	0.00304 (0.00232 to 0.00393)
HR (95% CI), P-value^§^	1.038 (0.726 to 1.483), 0.58	
Vaccine efficacy (VE)^§§^, Point estimate (95% CI)	−0.038 (−0.483 to 0.274)	
Initial conversion proportion (95%CI)^‡^	0.1550 (0.1310 to 0.1790)	0.1472 (0.1234 to 0.1711)
Person time accrued (months)^¶^	19695.08	19370.78
Overall initial conversion incidence rate (95% CI)^¶¶^	0.0069 (0.0057 to 0.0081)	0.0065 (0.0054 to 0.0077)

* All analyses were performed in the modified intention-to-treat (ITT) population unless otherwise indicated. A QFT (QuantiFERON-TB Gold Plus In-tube assay) conversion was defined as a change from negative (<0.35 IU per milliliter) on Day 71 (or first visit after missed Day 71 visit) to positive (≥0.35 IU per milliliter).

† Participants who had primary QFT conversion from QFT-negative to QFT-positive and sustained positive QFT tests at both 3- and 6-months after initial conversion.

‡ Proportion was calculated as converters/N. 95% CI were calculated based on the Miettinen and Nurminen method without stratification.^16^

¶ Person Time was calculated as time to conversion if observed, or the censored time if conversion was not observed.

¶¶ Incidence rate was calculated using person time as the denominator. Exact confidence interval (CI) was based on a Poisson process with constant intensity

§ Hazard ratio (HR) was calculated based on stratified Cox proportional hazards model with sex and age groups (10-11 years old, 12-14 years old, and > 14 years old) as stratification variables. One-sided P-value was calculated based on log-rank test, stratified by sex and age group.

§§ Vaccine efficacy (VE)=1-HR (BCG/Placebo); VE (lower limit) = 1 – HR (CI upper limit), VE (upper limit) = 1 – HR (CI lower limit)

†† Participants with first QFT conversion from a negative to positive test result at or after Day 71 (if Day 71 visit was missed), irrespective of change from positive to negative at Day 84 or 6-month visit

**Table 2.** Incidence of adverse events (AEs)

Variable	BCG (N=918)		Placebo (N=917)	
	*n/N (%)*	*95% CI* ^§^	*n (%)*	*95% CI* ^§^
Solicited AEs*,**				
Any injection site symptom	712/915 (77.8)	75.0, 80.4	349/915 (38.1)	35.0, 41.3
Any general body symptom	371/916 (40.5)	37.4, 43.7	314/915 (34.3)	31.3, 37.4
Use of medication Day 1-7 post vaccination	30/903 (3.3)	2.3, 4.6	27/899 (3.0)	2.0, 4.3
Injection site symptoms by severity, n/N (%)	Any	Severe/Life Threatening	Any	Severe/Life Threatening
	*n/N (%)*	*n/N (%)*	*n/N (%)*	*n/N (%)*
Swelling	657/912 (72.0)	2/912 (0.2)	248/910 (27.3)	1/910 (0.1)
Pain	381/914 (41.7)	17/914 (1.9)	148/915 (16.2)	6/915 (0.7)
Redness	349/912 (38.3)	0	111/911 (12.2)	0
Solicited systemic AEs by severity, n/N (%)	Any	Severe/Life Threatening	Any	Severe/Life Threatening
Tiredness	229/913 (25.1)	8/913 (0.9)	184/913 (20.2)	6/913 (0.7)
Headache	196/913 (21.5)	7/913 (0.8)	164/913 (18.0)	9/913 (1.0)
Stomach Problems	136/913 (14.9)	8/913 (0.9)	140/913 (15.3)	8/913 (0.9)
Fever	24/911 (2.6)	5/911 (0.5)	27/907 (3.0)	5/907 (0.6)
AE Categories^†^	n (%)	95% CI^§^	n (%)	95% CI^§^
Mild AEs	315 (34.3)	31.3, 37.4	90 (9.8)	8.0, 11.9
Moderate AEs	47 (5.1)	3.8, 6.7	28 (3.1)	2.1, 4.3
Severe AEs	5 (0.5)	0.2, 1.2	3 (0.3)	0.1, 0.9
Related AEs	273 (29.7)	26.8, 32.8	9 (1.0)	0.5, 1.8
Related, Severe	1 (0.1)	0, 0.5	0	
**Unsolicited non-serious** AEs	185 (20.2)	17.7, 22.8	117 (12.8)	10.7, 15.0
SAEs	3 (0.3)	0.1, 0.9	3 (0.3)	0.1, 0.9
SAEs with outcome of death	0		0	
Serious ADR¶	0		0	
AEs leading to premature trial discontinuation	0		0	
AEs of special interest	5 (0.5)	0.2, 1.2	0	
Most common (≥1%) unsolicited non-serious AEs in any group, n (%)				
Headache	36 (3.9)		27 (2.9)	
Injection-site pain	21 (2.3)		0	
Injection-site ulcer	20 (2.2)		0	
Pyrexia	12 (1.3)		3 (0.3)	
Oropharyngeal pain	9 (1.0)		4 (0.4)	
Upper respiratory tract infection	8 (0.9)		15 (1.6)	

* All analyses were performed in the safety population. MedDRA Version 26.0 was used for coding adverse events.

** Data are based on the AEs reported on the diary card from days 1 to 7. Participants were categorized under the highest severity experienced. Fever was defined as ≥38.0° C.

† AE data collection period: solicited AEs from Day1 through Day 7, unsolicited AEs from Screening through Day 28, SAEs from screening through Month 6, serious ADRs from Day 1 through end of trial. An AE with changing severity was counted only once under highest severity for the n and % values. A participant with multiple AEs within a system organ class or preferred term was counted only once.

¶ Serious adverse drug reaction (ADR): SAE assessed as related to trial intervention.

§ 95% CIs were calculated based on the conditional binomial Clopper-Pearson method with mid-p correction.
