# Supplementary material for: BCG Revaccination for the Prevention of Mycobacterium tuberculosis Infection
Source: N Engl J Med. Author manuscript; Available in PMC 2025 May 8. (PMC12061034; doi:10.1056/NEJMoa2412381)
Supplement: Supp [file NIHMS2068097-supplement-Supp.pdf]

Supplementary Appendix

Table of Contents

List of staff at trial sites **3**

Additional Methods **5**

Participant eligibility criteria 5

QFT Assessments 7

Safety and reactogenicity of BCG revaccination 7

Immunogenicity 8

Antigen-specific CD4 T-cell responses ***8***

WB-ICS assay ***9***

Statistical analysis 10

Sample size ***10***

QFT conversions ***10***

Immunogenicity ***11***

Supplementary Results **12**

Safety 12

Supplementary figures **13**

Figure S1: Cumulative event curve for initial QFT conversion using an assay threshold of 4.0 IU/mL (mITT population). mITT, modified intention-to-treat population; QFT, QuantiFERON-TB Test 13

Figure S2: Kinetics of antigen-specific CD4 T-cell responses expressing any combination of IL-2, IFN-γ, TNF, IL-22 and/or IL-17 (i.e. the total cytokine response) at D1, D29, D71 and D168 (month-6-visit) in each trial arm. 14

Figure S3: Kinetics of antigen-specific CD4 T-cell responses expressing Th1 cytokines (IL-2, IFN-γ, and/or TNF) or Th17/22 cytokines (IL-17 and/or IL-22) at D1, D29, D71 and D168 (month-6-visit) in each trial arm. 15

Figure S4: Kinetics of antigen-specific CD8 T-cell responses expressing Th1 cytokines (IL-2, IFN-γ, and/or TNF) at Day 1, 29, 71 and 168 (month-6-visit) in each study arm. 16

Supplementary Tables **17**

Table S1: Representativeness of Trial Participants ..... **Error! Bookmark not defined.**

Table S2: QFT positivity rate at screening 18

Table S3. Baseline characteristics (safety population) 18

Table S4: Sustained QFT conversion rate and vaccine efficacy point estimates by trial site (mITT population) 19

Table S5: Early QFT reversions (Safety Population) 19

Table S6: Initial QFT conversions using different interferon gamma (IFN-g) thresholds[†] 20

Supplementary references **22**

List of staff at trial sites

<u>Mbekweni, Paarl, WC</u>

Chrisna Andersen, Mia van Velden, Jeanne-Marie Myburgh, Debra Pote, Erica Bailey, WA Koegelenberg, Candise Hans, Eileen Fortuin, Thandolwethu Dada, Zeti Telite, Mziwoxolo Nqwede, Adri Holm, Nomthandazo Matolengwe, Noxolo Bonakele, Anine Theron

<u>Crossroads, Cape Town, WC</u>

Babalwa Khaya, Bonita Smitsdorff, Bukiwe Mnqebisa, Caro-Lee Saal, Cleon Ncayiya, Edith Kiribikiri, Elaine Sebastian, Ferial Mahed, Hlengiwe Mkhize, Jenipher Gelant, Julio Muller, Justine Stewart, Kgomotso Mocumi, Khadija Gool, Lindsay Jeffrey, Litha Gogo, Llewellyn Fleurs, Lulama Maqungo, Lulu Nair, Michaela Kingwill, Michelle Van Der Merve, Mluleki Nompondwana, Mustafaa Maarman, Mzamo Ntanjana, Ndiseka Nashwa, Njabulo Shangase, Nobubele Mshudulu, Nocwaka Magobiane, Nokwayintombi Scotch, Noluxolo Mgxekeza, Nombeko Mpongo, Nombongo Mayekiso, Nontshukumo Ngqabe, Noxolo Mona, Nozicelo Mbiza, Ntombekhaya Zoneleni, Nwabisa Danster, Pam Makhamba, Pamela Dukwe, Priscilla Mvinjelwa, Ridley Howard, Samantha Geduld, Sibusiso Gumede, Sihle Dyanti, Tamara Honono Thandokazi Tofile, Thembakazi Daki, Thembisile Yola, Thobeka Coba, Valerie Mlotshwa, Winnifred Gumula, Xolani Gxako, Zandile Mkhize, Jabulisile Zuma, Nomvuselelo Tshongoyi, Ebrahim Jacobs, Abigail van der Linde, Baphiwe Tose, Noluthano Khiya, Amina Ismail, Shakeera Arnolds, Sithembiso Mbhele, Junaid Rawoot, Zanoxolo Mthethi, Sithembiso Mbhele, Jaco Horak, Philippa Macdonald, Scott Mahoney, Lusanda Yekani, Nazmie Pearce, Janice Groenewald, Theodorah Rirhandzu Ndzhukule, Gakiema Malan, Steven Innes, Khaya Vakala, Lungile Mafilika, Yonela Mathiso, Llewellyn Fleurs, Steven Innes, Lindsay Jeffery, Kgomotso Mocumi, Caro-Lee Saal, Justine Stewart

Worcester, WC

Henrich Strumper, Mechia Gerber, Sonia Stryers, Beaulah Speckman, Bongani Diamond, Ilse Davids, Lauren Mactavie, Ruwiyda Jansen, Nambitha Nqakala, Nompumelelo Cetwayo, Xoliswa Kelepu, Roxane Herling, Likhaya Sizani, Julia Amsterdam, Nicolette Tredoux, Marwou de Kock, Michelle Williams, Claire Imbratta, Lungisa Jaxa, Lauren Cruywagen, Constance Schreuder, Munyaradzi Musvosvi, Nicole Bilek,Yolundi Cloete, Mzwandile Erasmus, Onke Nombida, Hadn Africa, Marcia Steyn, Habibullah Valley, Sandisiwe Mangali, Hlengiwe Nkambule, Lebohang Makhethe

Durban, KZN

Victress Busisiwe Ntsalaze, Gillian Dorse, Nonzwakazi Ntombela, Cailin Ball, Senzo Ndlovu, Zanele Gwamanda, Dudu Dlamini, Phindile Sing, Senzo Hlathi, Goodness Gumede, Atika Moosa, Lindiwe Ngcobo, Sindiswa Msomi, Kynesha Moopanar and Kandrelle Naidoo

Johannesburg, GP

Elizea Horne, Faeezah Patel, Tiffany Seef. Othusitse Segalo, Jeanne Coetzee, Muneerah Khan, Israel Mzizi, Rorisang Komane, Hamisha Soma-Kasiram, Vimbisai Mukoko, Saajida Akhalwaya, Stela Mthombeni, Thando Ntuli, Mapule Moloi, Sanele Nkosi, Angela Oosthuizen, Janet Grab, Masebole Masenya

Additional Methods

Participant eligibility criteria

Participants were included in the trial if they met all the following criteria:

1. ≥ 10 years and ≤ 18 years on trial Day 1
2. General good health, confirmed by medical history and physical examination
3. Vaccinated with BCG at least 5 years ago, documented through medical history or by presence of healed BCG scar
4. Tested QFT negative at screening
5. For female participants: not pregnant and agreed to avoid pregnancy throughout the first 12 months of the trial. Women physically capable of pregnancy must agree to use an acceptable method of avoiding pregnancy during this period. Acceptable methods of avoiding pregnancy included sexual abstinence (not engaging in sexual intercourse), a confirmed sterile partner, or at least 2 contraception methods from the following list: male or female condom, diaphragm, intrauterine devices (IUDs), hormonal contraceptive (oral, injection, transdermal patch, or implant)
6. Agreed to stay in contact with the trial site for the duration of the trial, provided updated contact information as necessary, and had no current plans to move from the trial area for the duration of the trial
7. Capable of giving signed informed consent/assent and completed the written informed consent/assent process

Participants were excluded if they had one of the following:

1. Acute illness or body temperature ≥37.5^{o}C on trial Day 1. This was a temporary exclusion for which the subject may be re-evaluated.

2. History or evidence of any clinically significant disease, including severe eczema and severe asthma, or any acute or chronic illness that might affect the safety, immunogenicity, or efficacy of trial vaccine in the opinion of the investigator
3. Any current medical, psychiatric, occupational, or substance abuse problems that, in the opinion of the investigator, will make it unlikely that the participant will comply with the protocol
4. History of autoimmune disease or allergic disease that is likely to be exacerbated by any component of the trial vaccine, or latent *Mtb* infection
5. History or evidence of active TB disease, or of any past or present possible immunodeficiency state including, but not limited to, any laboratory indication of HIV-1 infection
6. History of treatment for active TB disease or received investigational TB vaccine at any time prior to trial Day 1
7. Received a tuberculin skin test within 6 months prior to Day 1
8. Received immunosuppressive treatment, e.g., chemotherapy, biologics or radiation therapy, or used immunosuppressive medication (daily steroid equivalent of ≥5mg prednisone) within 42 days before trial Day 1.
9. Received immunoglobulin or blood products within 42 days before trial Day 1
10. Planned administration/administration of a licensed vaccine in the period starting 28 days before and ending 28 days after trial Day 1
11. Received any investigational drug therapy or investigational vaccine within 180 days before Day 1, or planned participation in any other clinical trial using investigational product during the trial period
12. Laboratory values from the most recent blood collected prior to randomization outside the normal range that are suggestive of a disease state

13. Urinalysis abnormality greater than Grade 1 on the Toxicity Scale (with the exception of
14. hematuria in a menstruating female), or urinalysis abnormality judged clinically significant by the investigator
15. Shared residence with an individual who is receiving TB treatment or with someone who is known to have incompletely treated TB. E.g., Xpert MTB/RIF assay-positive, PCR-positive, culture-positive, smear-positive TB, or clinically diagnosed unconfirmed TB.
16. Female participants currently pregnant or lactating/nursing; or positive serum pregnancy test during screening or on Day 1, prior to vaccination, or planning a pregnancy within the first 12 months after trial intervention
17. A child in care defined as a child who is under the care (control or protection) of an agency, organization, institution or entity by the courts, the government body, acting in accordance with powers conferred in them by law or regulations, or a child who is cared for by foster parents or living in a care home or institution, provided that the arrangements falls within the definition above

QFT Assessments

QFT assays were performed at screening and Day 71 (D71) or at the first in-person visit after D71 (if D71 visit was missed or if the D71 result was not available). A 70-day wash-out period was used to identify participants who had become infected with *Mtb* just prior to or soon after enrollment. Due to COVID pandemic measures, the D71 visit was missed for many participants and therefore a negative QFT was required at or after D71.

Safety and reactogenicity of BCG revaccination

Participants received a diary card on day 1 to record the duration and intensity of solicited local and systemic AEs, as well as unsolicited AEs. Solicited AEs were recorded for 7 days post vaccination and unsolicited AEs through 28 days post vaccination. Solicited AEs ongoing

beyond day 7 post vaccination were recorded as unsolicited AEs from Day 8. All serious AEs (SAEs) and AEs of special interest (AESIs) were reported through 6 months and serious adverse drug reactions (serious ADRs) through the end of the trial.

Immunogenicity

A subset of the first 80 10- and 11-year-old participants enrolled in Worcester were included in the immunogenicity cohort. Whole blood was collected at the Day 1, 29, 71, and Month 6 visits, and were processed using whole blood intracellular staining (WB-ICS) assay to measure antigen-specific immune responses. Children living in care and participants who converted to QFT positive at Day 71 were excluded from the per protocol immunogenicity (PPI) analyses but were followed up for safety and efficacy until end-of-study. A total of 12 participants were excluded from PPI analysis.

Antigen-specific CD4 T-cell responses

Antigen-specific CD4 and CD8 T-cell responses were evaluated using WB-ICS assay as previously described[1]. Briefly, whole blood was collected in sodium heparin tubes and stimulated within 75 min with BCG peptide pool (1µg/mL), BCG SSI (1 x 10^6 CFUs/mL) or left unstimulated in the presence of co-stimulatory antibodies (anti-CD28 and CD49d, at 0.25 µg/mL each, BD) for 12 hours at 37°C. After 7 hours of stimulation, Brefeldin A (10µg/mL, Sigma Aldrich) was added, and the blood was incubated for another 5 hours at 37°C. Erythrocytes were lysed and leukocytes fixed using FACS lysing solution (BD). For flow cytometry, leukocytes were stained using the monoclonal antibody panel listed in Supplementary Table S6 below. Acquisition was performed in batches that included samples collected from the same participant (i.e., Days 1, 29, 71, and 168) to minimize within-participant variability.

WB-ICS assay

Stained samples were acquired on a BD FACSymphony™ A5 Cell Analyzer flow cytometer configured with 5 lasers. Compensation and transformation, including the removal of inconsistent events during sample acquisition were manually performed using FlowJo version 10 (BD). A qualified operator performed gating for all FCS files using a consistent template. In parallel, as a quality control strategy, compensated FCS files in CSV-format were exported and analyzed using an OpenCyto-based automated gating method implemented in R version 4.1.0, to determine frequencies of cytokine-producing CD4 and CD8 T cells. Flow cytometry plots for samples with outlier frequencies of lymphocytes, CD3 cells, and cytokine-expressing cells were manually inspected to rule out atypical staining patterns.

The responder status for each participant was derived by determining whether a sample had significantly higher counts of cytokine-expressing T cells in the stimulated, relative to the unstimulated, condition. This was done by computing p-values using Fisher's exact test and participants with samples yielding P-values <0.01 were defined as responders. For background subtraction all possible cytokine co-expressing CD4 or CD8 T cell subsets identified by Boolean logic for cytokine gates, and cytokine-positive cell frequencies in unstimulated samples were subtracted from their corresponding antigen-stimulated cytokine-positive cell subsets. To avoid biasing responses to the positive, the distribution of background-subtracted frequencies around 0 was computed and the 80% CI for all negative values after background subtraction was derived. The absolute value of the lower (negative) 80% CI was then applied as a threshold for positive values, and all values less than this threshold were set to 0.

Statistical analysis

Sample size

The proposed sample size per group was determined based on targeting relatively high probability (~80%) of obtaining 118 sustained conversion events within 3.5 years of study start, assuming one year to complete enrolment, a primary endpoint incidence rate of 5.6% per person year and VE of 45%. Since ~12% of the participants enrolled were expected to be 10- or 11-year-old adolescents, with limited information on incidence rate and potential vaccine efficacy, we increased the sample size by 12% in each group resulting in a final proposed sample size of 900 participants per group.

QFT conversions

The primary endpoint was analyzed based on conversions to a positive result (IFN-γ ≥0.35 IU per milliliter) on the QuantiFERON-TB Gold Plus In-tube assay, with sustained positive results at both 3- and 6- months after initial conversion. Analyses were conducted in the modified intention-to-treat population, which required participants to have received treatment and to have a negative test result (IFN-γ <0.35 IU per milliliter) on Day 71 (or first visit after missed Day 71 visit). Statistics used to summarize this endpoint are presented in Table 1 and included the proportion or participants who had an initial or sustained conversion event, calculated as converters/N as well as the number of reversions. Initial conversion was defined as the first QFT conversion from a negative to positive test result at or after Day 71 (if Day 71 visit was missed), irrespective of change from positive to negative at day 84 or 6-month visit. Participants who had an initial conversion followed by a negative QFT result at either the Day 84 or Month 6 visit were considered reversions. Participants with indeterminate QFT results or missed a visit within the 6-month window following initial conversion were not evaluable for reversion. 95% CI for the

proportion of sustained converters were calculated based on the Miettinen and Nurminen method without stratification.[2]

For time to event analyses and rate calculations, person time was calculated as time to conversion if observed, or the censored time (end of follow up) if conversion was not observed. The incidence rate was calculated using person time as the denominator with an exact CI based on a Poisson process with constant intensity. Vaccine efficacy (VE) was defined as 1-Hazard Ratio (BCG/Placebo), where the hazard ratio (HR) was calculated based on stratified Cox proportional hazards model with sex and age groups (10-11 years old, 12-14 years old, and > 14 years old) as stratification variables. The proportional hazards assumption was valid per the supremum test p-value of 0.74. The one-sided P-value was calculated based on the non-parametric log-rank test, stratified by sex and age group. The CI for vaccine efficacy was calculated using the CI for the HR, such that VE (lower limit) = 1 – HR (CI upper limit), VE (upper limit) = 1 – HR (CI lower limit).

Multiplicity

To account for multiple testing, a step-down approach was planned to preserve the overall Type 1 error at a one-sided 2.5% for the primary, secondary and final analyses of the primary endpoint. Specifically, if the one-sided p-value associated with the primary hypothesis of BCG VE relative to placebo was less than or equal to 0.025, the durability of the VE after all participants had 36 months of follow-up would then be formally assessed. If both the primary hypothesis and the assessment at 36 months of follow-up were significant, then the assessment at 48 months could be formally assessed. Once a hypothesis was rejected, no further testing would be formally permitted. No other multiplicity adjustments were planned.

Immunogenicity

Immunogenicity figures were generated in GraphPad Prism (https://www.graphpad.com) and AUC was computed using the Trapezoid method for each participant.

Supplementary Results

Safety

Trial intervention-related AEs of any grade were observed in 273 (29.7%) participants in the BCG group and 9 (1.0%) in the placebo group**.** One participant in the BCG group had a related severe AE of headache. Five (0.5%) participants in the BCG group had AEs of special interest (injection site abscess in 4 and bacterial lymphadenitis in 1). Three participants (0.3%) in each group had SAEs. None of them were assessed as related. (BCG group: sinusitis and subperiosteal abscess in 1 participant; intentional overdose in 1, and fibula fracture, tibia fracture and traumatic hemothorax in 1; placebo group: appendicitis in 1 participant, meningitis and tuberculous in 1, and intentional overdose in 1).

Supplementary figures

Figure S1: Cumulative event curve for initial QFT conversion using an assay threshold of 4.0 IU/mL (mITT population). mITT, modified intention-to-treat population; QFT, QuantiFERON-TB Test

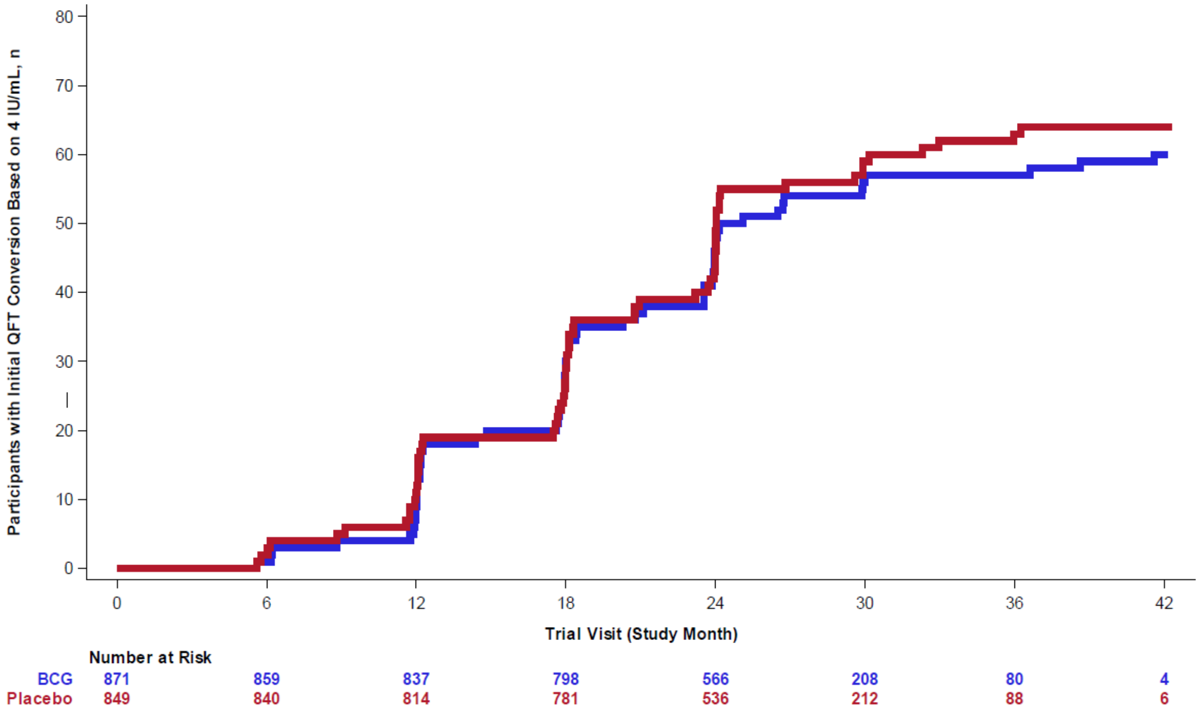

Figure S2: Kinetics of antigen-specific CD4 T-cell responses expressing any combination of IL-2, IFN-γ, TNF, IL-22 and/or IL-17 (i.e. the total cytokine response) at D1, D29, D71 and D168 (month-6-visit) in each trial arm.

Frequencies of antigen-specific CD4 T cells expressing any combination of IFN-γ, TNF, IL-2, IL-17, and IL-22 after stimulation with BCG and measured by whole blood intracellular cytokine staining assay in participants receiving placebo or BCG. (A) Longitudinal trajectories of CD4 T-cell responses in the BCG (blue) and placebo (red) group at the indicated time-points. (B). Fold change in CD4 T-cell responses between D71 and D0, for the two study arms. The dots represent the estimated median while the error bars represent the 95% CI.

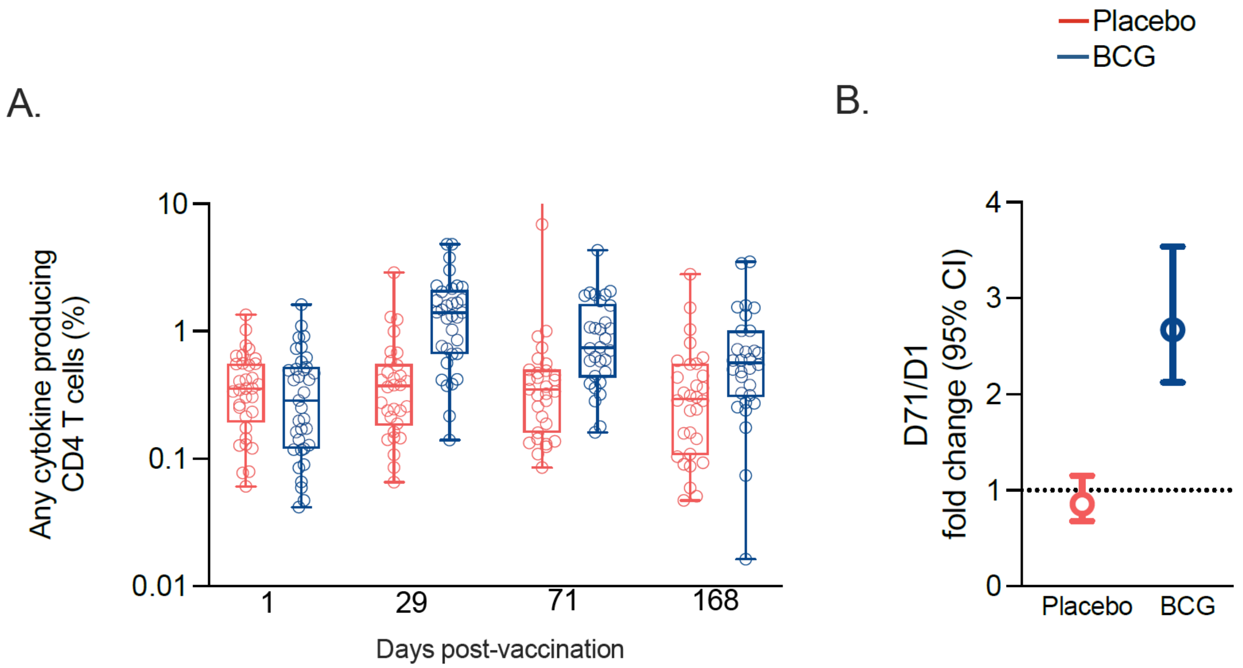

Figure S3: Kinetics of antigen-specific CD4 T-cell responses expressing Th1 cytokines (IL-2, IFN-γ, and/or TNF) or Th17/22 cytokines (IL-17 and/or IL-22) at D1, D29, D71 and D168 (month-6-visit) in each trial arm.

Frequencies of antigen-specific CD4 T cells expressing IL-2, IFN-γ, and/or TNF (panels A through D), and IL-17 and/or IL-22 (panel E though H) after stimulation with BCG and measured by whole blood intracellular cytokine staining assay in participants receiving placebo or BCG. (A and E) Longitudinal trajectories of CD4 T-cell responses in the BCG group (blue) and the placebo group (red) at the indicated time-points. (B and F) AUC for antigen-specific CD4 T-cell responses during the first 168 days after vaccination. Each dot represents an individual participant. (C and G) Comparison of antigen-specific CD4 T-cell responses at the indicated time-points. Horizontal lines within each box represent medians, boxes represent IQRs and whiskers the minimum and maximum values. (D and G). Fold change in CD4 T-cell responses between D71 and D0. The dots represent the estimated median while the error bars represent the 95% CI.

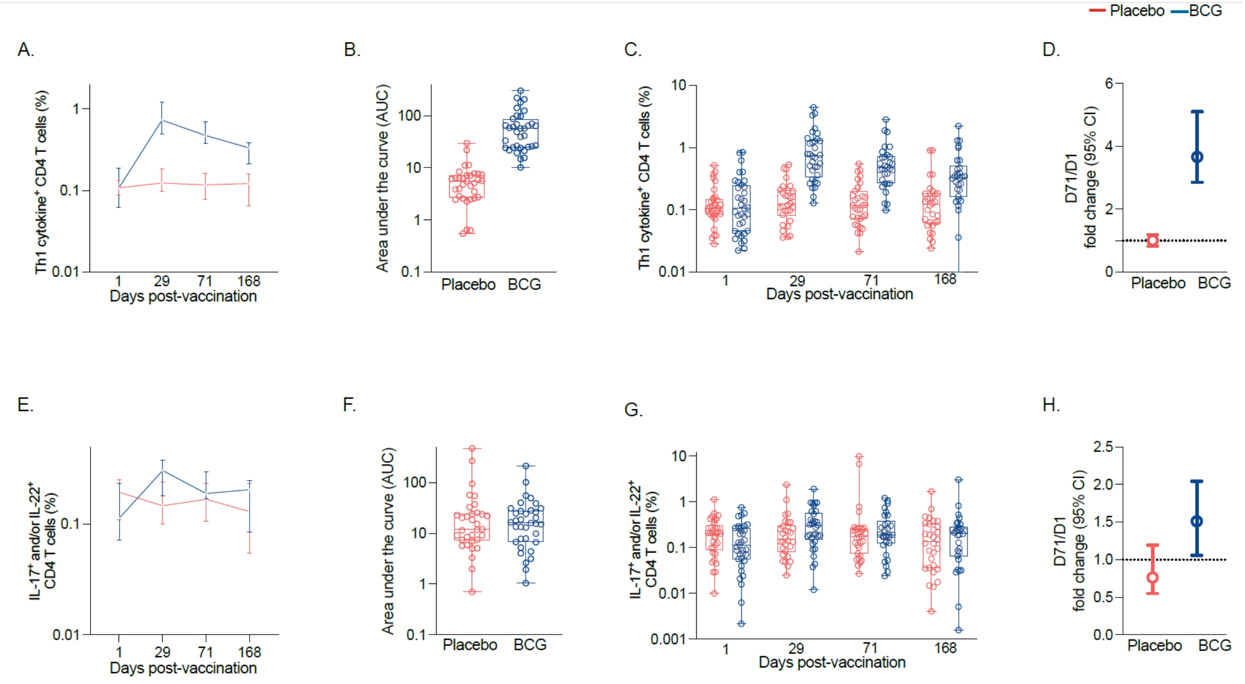

Figure S4: Kinetics of antigen-specific CD8 T-cell responses expressing Th1 cytokines (IL-2, IFN-γ, and/or TNF) at Day 1, 29, 71 and 168 (month-6-visit) in each study arm.

Frequencies of antigen-specific CD8 T cells expressing any combination of IFN-γ, TNF, and/or IL-2 after stimulation with BCG and measured by whole blood intracellular cytokine staining assay in participants receiving placebo (red) or BCG (blue). (A) Longitudinal trajectories of CD8 T-cell responses in the BCG and placebo group at the indicated time-points. (B) AUC for antigen specific CD8 T-cell response during the first 168 days after vaccination. Each dot represents an individual participant. (C) Comparison of antigen-specific CD8 T-cell responses at the indicated time-points between BCG and placebo recipients. Horizontal lines within each box represent medians, boxes represent IQRs and whiskers the minimum and maximum values.

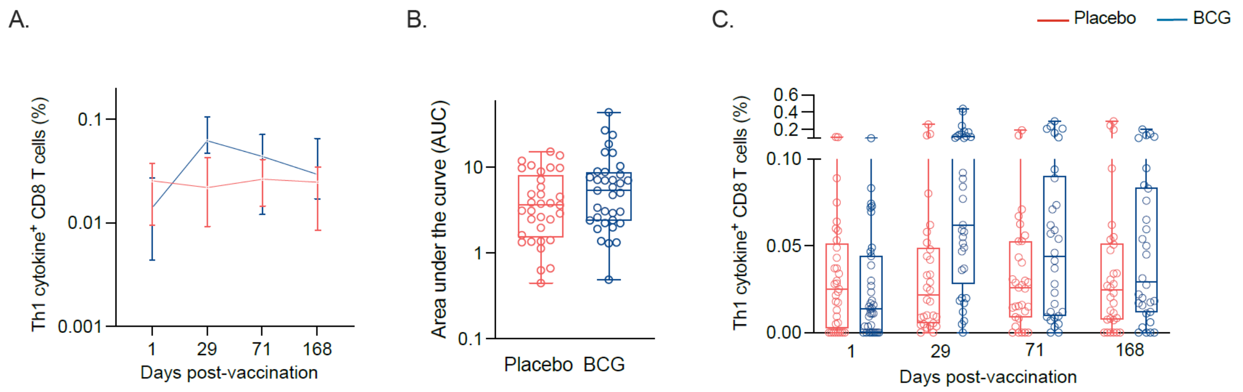

Supplementary Tables

Table S1: Representativeness of Trial Participants

Category	Example
Disease, problem, or condition under investigation	Infection with M. tuberculosis and progression to active pulmonary TB
Special considerations related to	
Sex and gender	Prevalence of *Mtb* infection is similar in male and female adolescents, but TB disease is more common in males than females (ratio of 1.5 to 1)
Age	In high-risk communities in South Africa, *Mtb* infection prevalence increases steeply from the age of 12 years and then levels off in the third or fourth decade of life.
Race or ethnic group	In South Africa, people of black African descent and people of mixed racial ancestry are at much higher risk than people of European or Asian descent, largely as a result of apartheid policies that led to forced segregation and inequity.
Geography	In South Africa, prevalence of *Mtb* infection by age and incidence of TB differ greatly across the country. Heterogeneity is also seen at province level, but also at district and sub district level, and by neighborhoods.
Other considerations	TB is a disease of poverty. The prevalence and incidence of TB are highest in populations who live in densely populated communities that suffer from poverty. Malnutrition, exposure to particulates, underlying lung disease, diabetes, alcohol use, and frequent exposure to people with active pulmonary TB increase the risk of infection, re-infection, and progression to TB disease.
Overall representativeness of this trial	The participants in the present trial were enrolled from communities at very high risk of *Mtb* infection and TB disease. The clinical trial sites in Mbekweni / Paarl and Crossroads / Cape Town are located in majority Xhosa-speaking townships and enrolled participants who live near the site. The Worcester site recruited participants attending various schools located within a 40 km radius of the research center in the Breede Valley. Most participants identified as South African Coloured or Mixed Race. The site in Johannesburg enrolled participants from inner city Johannesburg as well as from several townships in or near Johannesburg. The population enrolled were Black Africans, speaking isiZulu, SeSotho as well as English. The site in Durban enrolled participants from communities living within a 50 km radius of the research clinic located in the Durban city center. The participants in the present trial appear to be representative of the high-risk communities they live in. Sex was reported by the participants. Options were Female and Male. Participants reported their ethnicity (Hispanic or Latino, Not Hispanic or Latino, Not Reported, Other) and race (Black, White, South African Coloured, Mixed Race, Asian Indian, Asian, Other). The demography form also asked the month and year of birth, which school the participant attends and region/province of the school.

Table S2: QFT positivity rate at screening

Clinical trial Site	QFT positive rate, % (n/N)
Overall	37 (1219/3333)
Mbekweni, Paarl, WC	38 (355/938)
Crossroads, Cape Town, WC	38 (257/674)
Worcester, WC	40 (452/1140)
Durban, KZN	29 (94/323)
Johannesburg, GP	24 (61/258)

Table S3. Baseline characteristics (safety population)

	BCG (N=918)	Placebo (N=917)	Total (N=1835)
Site, n			
Mbekweni, Paarl, WC	265	263	528
Crossroads, Cape Town, WC	181	183	364
Worcester, WC	293	290	583
Durban, KZN	88	92	180
Johannesburg, GP	91	89	180
Median age (range), years	13 (10-18)	13 (10-18)	13 (10-18)
Age groups, n (%), years			
10-11	82 (8.9)	84 (9.2)	166 (9.0)
12-14	597 (65.0)	590 (64.3)	1187 (64.7)
>14	239 (26.0)	243 (26.5)	482 (26.3)
Female, n (%)	474 (51.6)	469 (51.1)	943 (51.4)
Race, n (%)			
Black African	729 (79.4)	735 (80.2)	1464 (79.8)
Asian Indian	0 (0.0)	1 (0.1)	1 (0.1)

Mixed Race	63 (6.9)	61 (6.7)	124 (6.8)
South African Coloured	125 (13.6)	120 (13.1)	245 (13.4)
White	1 (0.1)	0 (0.0)	1 (0.1)
Median body-mass index (range)	19.5 (13.0 to 43.8)	19.4 (12.8 to 43.0)	19.4 (12.8 to 43.8)

Table S4: Sustained QFT conversion rate and vaccine efficacy point estimates by trial site (mITT population)

Sites	Sustained converters, n/N (%)†		Vaccine efficacy point estimate (95%CI)‡
	BCG	Placebo	
Mbekweni, Paarl, WC	29/256 (11.3)	22/248 (8.9)	-0.2841 (-1.238 to 0.2633)
Crossroads, Cape Town, WC	6/170 (3.5)	11/170 (6.5)	0.4625 (-0.4651 to 0.8028)
Worcester, WC	25/273 (9.2)	22/258 (8.5)	-0.1016 (-0.9560 to 0.3796)
Durban, KZN	2/83 (2.4)	0/86	NE
Johannesburg, GP	0/89	4/87 (4.6)	NE

†Participants who had primary QFT conversion from QFT-negative to QFT-positive and sustained positive QFT tests at both 3- and 6-months after initial conversion.

‡Vaccine efficacy (VE)=1-HR (BCG/Placebo); VE (lower limit) = 1 – HR (CI upper limit), VE (upper limit) = 1 – HR (CI lower limit); NE, not estimable

Table S5: Early QFT reversions (Safety Population)

	BCG (N=871)	Placebo (N=849)
Reversions (n) at post-conversion Day 84†	37	33
Reversions (n) at post-conversion Month 6¶	12	9
Total Reversions (n) and Reversion Rate [95% CI]*	49 (0.36 [0.29 to 0.45])	42 (0.34 [0.26 to 0.42])

†Participants with an initial positive QFT result (after Day 71) followed by a negative QFT result at post-conversion Day 84.
¶Participants with an initial positive QFT result (after Day 71) followed by a positive QFT result at post-conversion Day 84 and a negative result at post-conversion month 6 assessment.
*95% CI was calculated based on the conditional binomial Clopper-Pearson method with mid-p correction

Table S6: Initial QFT conversions using different interferon gamma (IFN-g) thresholds†

IFN-g threshold (IU/mL)	BCG (N=871)		Placebo (N=849)	
	Initial QFT converters, n (%)	Proportion of Initial QFT conversions (95% CI)*	Initial QFT converters, n (%)	Proportion of Initial QFT conversions (95% CI)*
0.36	136 (15.6)	0.1561 (0.1320, 0.1802)	119 (14.0)	0.1402 (0.1168, 0.1635)
0.4	130 (14.9)	0.1493 (0.1256, 0.1729)	119 (14.0)	0.1402 (0.1168, 0.1635)
1.0	97 (11.1)	0.1114 (0.0905, 0.1323)	94 (11.1)	0.1107 (0.0896, 0.1318)
2.0	83 (9.5)	0.0953 (0.0758, 0.1148)	82 (9.7)	0.0966 (0.0767, 0.1165)
3.0	69 (7.9)	0.0792 (0.0613, 0.0972)	72 (8.5)	0.0848 (0.0661, 0.1035)
4.0	60 (6.9)	0.0689 (0.0521, 0.0857)	64 (7.5)	0.0754 (0.0576, 0.0931)
5.0	54 (6.2)	0.0620 (0.0460, 0.0780)	55 (6.5)	0.0648 (0.0482, 0.0813)
6.0	45 (5.2)	0.0517 (0.0370, 0.0664)	53 (6.2)	0.0624 (0.0462, 0.0787)
7.0	33 (3.8)	0.0379 (0.0252, 0.0506)	38 (4.5)	0.0448 (0.0308, 0.0587)
10.0	0	0	0	0

†Participants who had the first QFT conversion after Day 71 based on the respective IFN-γ thresholds. *95% CIs were based on the Miettinen and Nurminen method without stratification

Table S7. Flow cytometry antibody panel for the whole blood ICS assay

Lineage/ function	Specificity	Fluorochrome	Clone	Manufacturer, Cat #	Volume*
T cells	CD8	BV510	SK1	BD, 563919	0.5
Th17 cytokine	IL-17A	BV650	N49-563	BD, 563746	2
T cells	CD4	BV786	SK3	BD, 563877	0.3
Th1 cytokine	IL-2	FITC	5344.111	BD, 340448	5
Th22 cytokine	IL-22	PE	22URTI	eBiosciences, 12-7229-42	2
Th1 cytokine	TNF	PE-Cy7	MAb11	eBiosciences, 25-7349-82	0.05
Th1 cytokine	IFN-g	AF700	B27	BD, 557995	0.1
T cells	CD3	APC-H7	SK7	BD, 560176	0.06
Differentiation	CCR7	PE-CF594	150503	BD, 562381	1
Differentiation	CD45RA	SBUV575	F8-11-13	Bio-Rad, MCA885BUV575	2
Function	CD153	APC	116614	WhiteSci, FAB1028A	5
MAIT cells	CD161	BUV737	DX12	BD, 748948	1
MAIT cells	TRAV1.2	BV605	3C10	BL, 351720	1.2
NK cells	CD16	BV805	3G8	BD, 569165	0.5
NK cells	CD56	BV711	HCD56	BL, 318336	2
gd T cells	gdTCR	BV421	B1	BL, 331218	1.5
Activation	HLA-DR	BV750	L243	BL, 307672	2

* µL/100µL of staining volume.

Supplementary references

1. Kagina BM, Mansoor N, Kpamegan EP, Penn-Nicholson A, Nemes E, Smit E, et al. Qualification of a whole blood intracellular cytokine staining assay to measure mycobacteria-specific CD4 and CD8 T cell immunity by flow cytometry. J Immunol Methods. 2015;417:22-33.
2. Miettinen O, Nurminen M. Comparative analysis of two rates. Stat Med 1985;4(2):213-26. DOI: 10.1002/sim.4780040211.
